# A 5-year change of knowledge and willingness by sampled respondents to perform bystander cardiopulmonary resuscitation in a metropolitan city

**DOI:** 10.1371/journal.pone.0211804

**Published:** 2019-02-07

**Authors:** Sungbae Moon, Hyun Wook Ryoo, Jae Yun Ahn, Jung Bae Park, Dong Eun Lee, Jung Ho Kim, Sang-chan Jin, Kyung Woo Lee

**Affiliations:** 1 Department of Emergency Medicine, School of Medicine, Kyungpook National University, Daegu, Republic of Korea; 2 Department of Emergency Medicine, College of Medicine, Yeungnam University, Daegu, Republic of Korea; 3 Department of Emergency Medicine, School of Medicine, Keimyung University, Daegu, Republic of Korea; 4 Department of Emergency Medicine, Daegu Catholic University Medical Center, Daegu, Republic of Korea; Azienda Ospedaliero Universitaria Careggi, ITALY

## Abstract

**Background:**

Nationwide and regional interventions can help improve bystander cardiopulmonary resuscitation (CPR) awareness, knowledge, and the willingness. Periodic community investigation will help monitor the effect. This study aimed to compare the experience of CPR education, CPR knowledge, and CPR willingness, during a 5-year interval.

**Methods:**

This is a pre and post study. Two surveys were done in February 2012 and December 2016. National and regional intervention including legislation promoting public involvement, standardizing CPR education programs, training CPR instructors, and installing supporting organizations were done at the period. In both surveys, respondents were selected via quota sampling in Daegu Metropolitan City and answered the survey through face-to-face interview. Respondents’ general demographic characteristics, CPR educational experience, CPR knowledge and CPR willingness were questioned.

**Results:**

Total of 2141 respondents (1000 in 2012, 1141 in 2016) were selected. The percentage of respondents who received CPR education itself and recent education were higher after intervention compared to before intervention (36.2% vs. 55.1%, 16.9% vs. 30.1%, respectively). Correct knowledge of performing CPR seems to be improved overall (1.6% vs. 11.7%, odd ratio 14.28, 95% confidence interval 5.68–35.94). However, less respondents were willing to perform CPR on strangers (54.5% vs 35.0%).

**Conclusion:**

Nationwide and regional interventions to promote bystander CPR and CPR education were associated with increased CPR education experience and improved correct CPR knowledge in performing bystander CPR. Willingness to perform bystander CPR on family did not increase significantly and CPR willingness to strangers was decreased. Additional legal and technological measures should be implemented to promote bystander CPR.

## Introduction

Sudden cardiac death remains one of the leading causes of death in developed countries[1] and is considered as a major burden to the population[2–3]. In the early 21^st^ century, there were approximately 600–700 non-traumatic out-of-hospital cardiac arrest (OHCA) cases annually in the Daegu Metropolitan City area; this number increased from 887 in 2012 to 1009 in 2016[4].

Bystander cardiopulmonary resuscitation (CPR) plays an important role in improving the survival rate and neurological outcomes of sudden cardiac arrest patients[5]. Various measures, including public education, can be implemented to achieve better outcome in performing bystander CPR[6–7]. Along with educational strategies, investigating public CPR awareness and CPR willingness to determine the associated factors is needed. It will also serve as a method of monitor and quality control of public CPR education[8].

**Fig 1:**
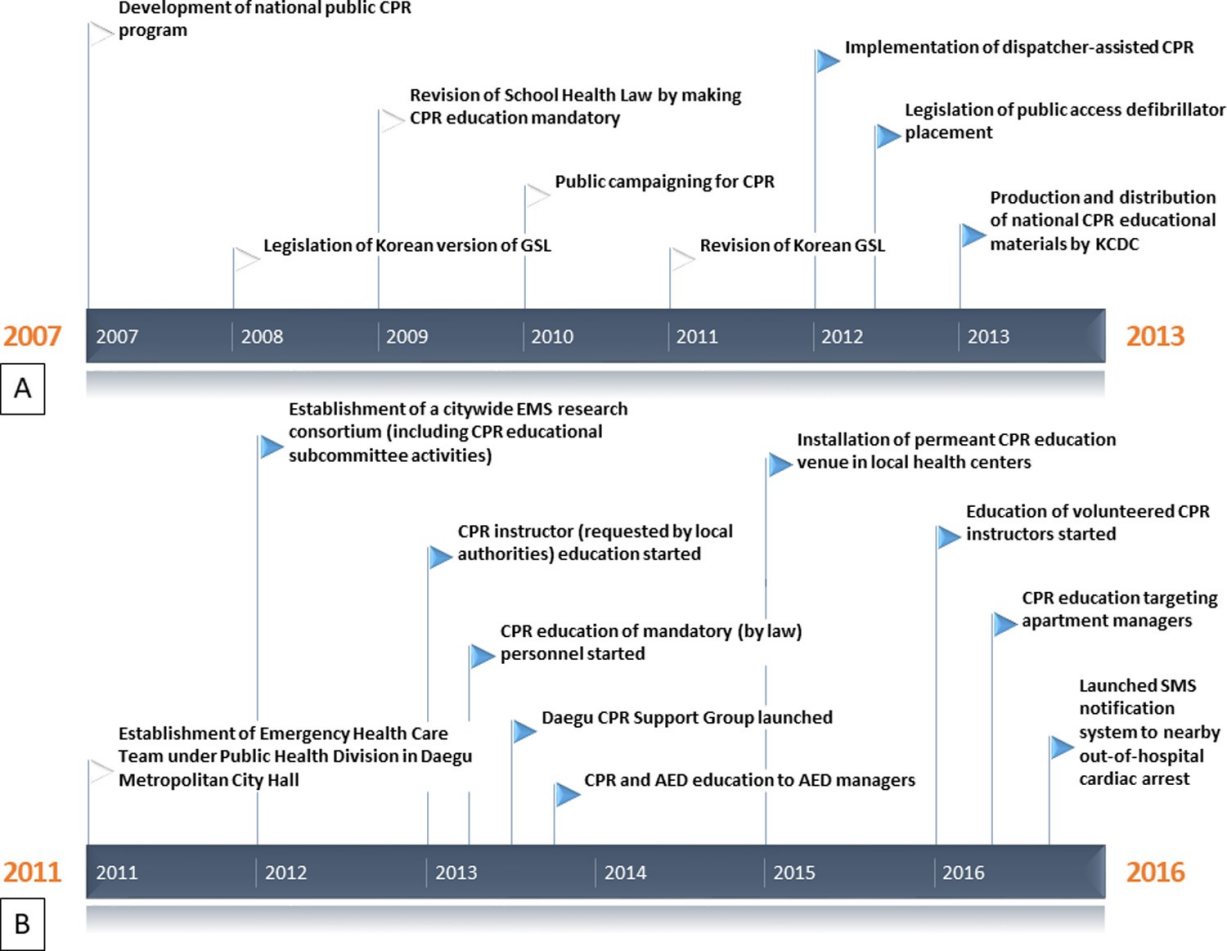
Timeline of nationwide and regional public interventions to improve bystander cardiopulmonary resuscitation. White triangles: interventions before the first survey. Filled triangles: interventions during two surveys. (A) Nationwide interventions. (B) Regional interventions. CPR, cardiopulmonary resuscitation; GSL, Good Samaritan Law; KCDC, Korean Center for Disease Control; EMS, emergency medical services; AED, automated external defibrillator.

In 2012, Son et al. conducted a study [9] which 1000 Daegu citizens participated in a survey regarding CPR willingness, CPR awareness, prior CPR education, confidence in CPR performance, interval from latest CPR education, and status of automatic external defibrillator (AED) training. National public CPR education program and public campaigning were already established before first survey was done. The survey was mainly intended to determine the effect of CPR education to bystander CPR willingness. After this study period, there were nationwide and regional public interventions, including legislation promoting public CPR engagement and AED installation, employment of certified personnel in public CPR education, production and distribution of standardized CPR educational material, and establishment of a citywide emergency medical service (EMS) research consortium. Interventions that occurred before and during the study period are portrayed in Fig 1.

The majority of previous studies about CPR willingness either did not focus on changes in regional scale [10–11] or did not represent the entire community population because they were concentrated on a certain age group[12–13]. Therefore, the aim of this study was to compare the rate of CPR education, correct CPR knowledge, and the willingness to perform CPR in a single metropolitan city, during a 5-year interval when national and regional interventions were carried out.

## Materials and methods

### Study design and subjects

Daegu is in the South-eastern part of the Korean peninsula. The city itself covers 883.63 km^2^ of the land area[14]. As of 2016, the city of Daegu had a population of 2,461,002[15]. In this region, the sudden cardiac arrest rate per 100,000 people was 48.3 in 2012, which increased to 53.7 in 2016[4].

This is a pre and post study. Two surveys were conducted in February 2012 (n = 1000) and December 2016 (n = 1141) among Daegu citizens aged ≥19 years who were selected by three-stage random sampling, which uses age, sex, and district population distribution, according to population census data. Selecting target population in second survey was done separately to the first survey population and was not affected by first survey population in any way. Before conducting the surveys, written consent was obtained from all participants after informing them about the purpose and objective of the surveys. In both surveys, 15 experienced interviewers employed in a private polling agency took part in interviewing the respondents. Before administering the actual surveys, interviewers underwent a pre-survey meeting session to standardize the interview method. Another educational session explaining the questionnaire and strictly detailed guideline was conducted to minimize human errors and discrepancies. After two training sessions, the interviewers visited each respondent at their homes to conduct face-to-face interviews using the structured questionnaires.

### Interventions

Nationwide interventions during the period included, but was not limited to, legislation of public access defibrillator program and composing standardized educational material for CPR, which corresponds to the latest resuscitation guidelines and legislating bills promoting CPR, protecting rescuers, and making CPR education mandatory at schools and workplaces. AED placement in certain public places and buildings was mandated by law and there was public and private funding available to promote AED installation. The overall number of AEDs in the Daegu area, excluding those deployed in medical facilities and EMS, was 203 in 2014, but 406 in 2015; at the end of 2016, the number was increased to 650[16]. Regional interventions focused on gathering, collaborating, and coordinating the competency of regional CPR education by standardized instructor training and instructor certification.

CPR education was the first target of the community intervention. The Daegu Emergency Medicine Research Group was founded in 2013. Emergency physicians, local government officials, paramedics, and concerned citizens gathered to propel developments on improving emergency medical service system in the Daegu area. One of the subcommittees was dedicated to popularizing and encouraging CPR education citywide by sponsoring periodic educational sessions by providing personnel, equipment, and feedback. The CPR educational subcommittee consisted of medical schools and was also associated with the local college of nursing and paramedics, joined by local office of education. The main activities of this subcommittee were to produce an instruction manual, develop different curriculums for first responders and by types of various layperson such as students, elderly, and children. The subcommittee, partnered with Daegu Metropolitan Office of Education, also helped setting up CPR education curriculum in local schools. We conducted a comprehensive recruitment of personnel who underwent public CPR education and CPR instructor courses, certified from either American Heart Association or Korean Association of Cardiopulmonary Resuscitation. Furthermore, educational materials (i.e., videos) were standardized using materials provided by the Korean Center of Disease Control[17].

### Questionnaire and variables

Board-certified emergency physicians in Daegu area drafted the initial questionnaires for both surveys. Selection and revision of the questionnaires were conducted via emergency physicians’ meeting. After pilot trials, researchers provided additional modifications.

Respondents’ general demographic characteristics, including age, sex and educational status were collected. Household income was also asked because it may be associated with CPR knowledge[12]. The participants were questioned regarding CPR awareness, knowledge, and willingness, and the status of CPR education. Among the questions about CPR knowledge, hand position, rate, and depth in chest compressions were included, as those questions were included in both surveys and three elements are commonly asked in other studies assessing CPR knowledge of general public[18–19]. Moreover, we also considered the possible barriers to perform bystander CPR and whether the interviewee was familiar with the current Korean Good Samaritans’ Law. Questionnaires for both surveys are presented in S1 Text.

### Statistical analysis

Participants were divided into groups according to the survey timing. Chi-square test was used to compare the general demographic characteristics, factors associated with CPR education, awareness, knowledge, and willingness between 2 groups. Logistic regression analysis was performed to investigate whether the survey timing, which will portray the result of interventions, affects in acquiring correct CPR knowledge and willingness to perform bystander CPR. Survey timing, general demographic factors which may affect CPR performance, interval from recent CPR education, and recognition of Korean Good Samaritans’ Law were used as independent variables. Statistical analysis was performed using IBM SPSS Statistics for Windows, Version 22.0 (IBM Corporation, Armonk, New York, USA). *P*-values <0.05 were considered statistically significant.

### Ethics

This study was approved by Kyungpook National University Hospital Institutional Review Board with a waiver of informed consent.

## Results

Total of 2141 respondents answered both surveys. The most common age group was 40–49 years in both the first and second surveys (23.3% and 21.0%, respectively). The percentage of respondents with a family history of cardiac disease was higher after intervention. Most people received college education (42.6% and 51.1%, respectively) and earned between 2 million to 3 million South Korean won (approximately 1900 to 2700 US dollars, 23.8% and 22.8%, respectively). The reason for a significant difference in age, educational status, and monthly income between groups was the change in the Daegu population census. More detailed results of the general demographic characteristics of respondents are described in Table 1.

**Table 1:** General demographic characteristics of respondents.

		1^st^ survey (n = 1000)	2^nd^ survey (n = 1141)	*P*
Sex	Male	480 (48.0)	545 (47.8)	0.914
	Female	520 (52.0)	596 (52.2)	
Age (years)	20’s^a^	178 (17.8)	216 (18.9)	0.01
	30’s	203 (20.3)	188 (16.5)	
	40’s	233 (23.3)	240 (21.0)	
	50’s	189 (18.9)	234 (20.5)	
	60’s	134 (13.4)	150 (13.1)	
	>70’s	63 (6.3)	113 (9.9)	
Fhx of cardiac disease	Yes	70 (7.0)	156 (13.7)	<0.001
Education	Junior high graduate or under	135 (13.5)	119 (10.4)	<0.001
	High school graduate	292 (29.2)	280 (24.5)	
	Undergraduate	103 (10.3)	108 (9.5)	
	College graduate	426 (42.6)	583 (51.1)	
	Master's or higher	31 (3.1)	51 (4.5)	
Household Income	<1 M (900 USD)	153 (15.3)	119 (10.4)	<0.001
(Monthly, KRW)	1 M–2 M (1900 USD)	202 (20.2)	208 (18.2)	
	2 M–3 M (2700 USD)	238 (23.8)	260 (22.8)	
	3 M–4 M (3600 USD)	213 (21.3)	218 (19.1)	
	4 M–5 M (4500 USD)	98 (9.8)	116 (10.2)	
	>5 M	53 (5.3)	154 (13.5)	

^a^: 19-year-olds included;

Fhx, family history; KRW, South Korean won; M, million; USD, United States dollar. Note that median household income in South Korea was 3.6M KRW (3300 USD) in 2012 and 4.4M KRW (3800 USD) in 2016.

The percentage of respondents who received CPR education was higher in 2^nd^ survey compared to 1^st^ survey (55.1% vs. 36.2%, respectively; Table 2). Furthermore, there were more respondents who received more recent (i.e., <2 years) CPR education in 2^nd^ survey compared to 1^st^ survey (30.1% vs. 16.9%, respectively). While the total number of CPR education received by the respondents did not change significantly, more respondents received AED training in 2nd survey compared to 1st survey (25.9% vs. 5.0%, respectively). The location where the respondents’ received CPR education is shown in survey questionnaire, as presented in S1 Text and S1 Table.

**Table 2:** Comparison of CPR education experience in both groups.

		1^st^ survey (n = 1000)	2^nd^ survey (n = 1141)	*P*
CPR education experience	Yes	362 (36.2)	629 (55.1)	<0.001
Number of CPR education	1	112 (11.2)	211 (18.5)	0.355
	2	119 (11.9)	214 (18.8)	
	3	47 (4.7)	99 (8.7)	
	>4	71 (7.1)	101 (8.9)	
Interval from recent CPR education	< 2 years	169 (16.9)	343 (30.1)	0.032
	> 2 years	182 (18.2)	275 (24.1)	
AED training included	Yes	50 (5.0)	295 (25.9)	<0.001

CPR, cardiopulmonary resuscitation; AED, automated external defibrillator.

Correct knowledge of performing CPR seems to be improved overall (1.6% vs. 11.7% in 1st and 2nd survey, respectively), as well as specific questions regarding compression hand position, rate, and depth. One noticeable result is that in both surveys, respondents were not aware of compression rate and depth as much as position. Compared to 1^st^ survey, more respondents were aware of AEDs (26.1% vs. 83.6%, respectively) and had claimed to know how to use an AED (4.8% vs 22.3%, respectively) in 2nd survey. (Table 3)

**Table 3:** Comparison of CPR knowledge, current awareness of AEDs, confidence, and willingness to perform CPR.

	1^st^ survey (n = 1000)	2^nd^ survey (n = 1141)	*P*
CPR knowledge (correct answers)			
Hand position	437 (43.7)	861 (75.5)	<0.001
Rate	42 (4.2)	216 (18.9)	<0.001
Depth	43 (4.3)	405 (35.5)	<0.001
All correct	16 (1.6)	134 (11.7)	<0.001
AED awareness			
“I know what an AED is”	261 (26.1)	954 (83.6)	<0.001
“I have seen AEDs in public places”	168 (16.8)	734 (64.3)	<0.001
“I know how to use an AED”	48 (4.8)	254 (22.3)	<0.001
Korean Good Samarian Law			
“I know about the law”	106 (10.6)	356 (31.2)	<0.001
Willingness			
CPR to family	723 (72.3)	856 (75.0)	0.073
CPR to stranger	545 (54.5)	399 (35.0)	<0.001
To use AED	400 (40.0)	561 (49.2)	<0.001

CPR, cardiopulmonary resuscitation; AED, automated external defibrillator.

Compared to 1st survey, an increased awareness on Korean version of Good Samarian Law (10.6% vs. 31.2%, respectively. Furthermore, more respondents were willing to use AED in 2^nd^ survey (49.2%) compared to 1^st^ survey (40.0%). Another noticeable result was that less respondents were willing to perform CPR on strangers (54.5% vs 35.0% in 1st and 2nd survey, respectively).

To investigate possible barriers in performing CPR, the questionnaires contained some questions about the specific reasons for these barriers. Although direct comparison and analysis were not possible due to changes in the answer selection method, majority of respondents selected lack of knowledge (316 vs. 375 respondents before and after intervention, respectively) and fear of harming the cardiac arrest victim (386 vs. 361 respondents before and after intervention, respectively) as the main reasons. Noticeably, fear of legal responsibilities related to bystander CPR, as a reason for unwillingness to perform CPR, seems to have doubled (106 vs. 201 respondents before and after intervention, respectively). Full table is available in S2 Table. Dispatcher-assisted CPR (DA-CPR) may help in overcoming such barrier and in our study, the majority of respondents in the second survey (54.7%) was willing to follow CPR instructions, if it was given by the dispatcher.

Table 4 shows factors related to the correct CPR knowledge and willingness to perform bystander CPR. Interventions related to CPR education during the study period, which are specified in Fig 1, and CPR education interval less than 2 years were important factors in acquiring better CPR knowledge (OR 14.28 and 3.03, respectively, after adjustments). For CPR willingness to strangers, while our intervention were predictors for less willing, CPR education interval and recognition of Korean Good Samarian Law yielded more willingness.

**Table 4:** Adjusted odd ratios and confidence intervals of factors associated with CPR knowledge and CPR willingness.

	Adjusted odd ratio (95% confidence interval)		
	CPR knowledge (all correctly answered)^a^	Willingness to provide CPR (family)^b^	Willingness to provide CPR (strangers)^b^
Group (2nd vs 1st)	14.28 (5.68–35.94)	0.62 (0.38–1.02)	0.18 (0.12–0.25)
Sex (female vs male)	0.81 (0.52–1.28)	0.90 (0.57–1.41)	0.85 (0.62–1.14)
Age (> 60s vs 19–59)	0.99 (0.40–2.40)	0.81 (0.37–1.76)	1.13 (0.65–1.97)
Fhx of cardiac disease	0.41 (0.16–1.07)	1.16 (0.55–2.45)	0.99 (0.61–1.60)
Education (UG vs HS)	1.45 (0.78–2.67)	0.66 (0.37–1.17)	0.93 (0.64–1.35)
Monthly income (KRW)			
<2 M (1900 USD)	Reference		
2 M–3 M (2700 USD)	0.81 (0.44–1.48)	1.17 (0.66–2.05)	1.26 (0.83–1.91)
>3 M	0.57 (0.33–0.99)	1.89 (1.10–3.25)	0.88 (0.61–1.27)
Interval from last CPR education (< 2 yr vs > 2 yr)	3.03 (1.83–4.99)	1.83 (1.17–2.87)	2.45 (1.82–3.31)
GSL recognition	N/A	1.57 (0.93–2.63)	1.68 (1.21–2.33)

^a^: adjusted for sex, age, family history of cardiac disease, degree of education, income, interval from recent CPR education;

^b^: also adjusted for recognition of Korean Good Samarian Law;

CPR, cardiopulmonary resuscitation; Fhx, family history; UG, undergraduate or higher; HS, high school graduate or under; KRW, South Korean won; M, million; USD, United States dollar; GSL, Korean Good Samarian Law.

## Discussion

Based on this study, we observed 2 substantial findings. Firstly, the percentage of the general population who received CPR education and knowing correct CPR knowledge increased during the 5-year study interval, meaning that interventions regarding public CPR education may have contributed to this result. The respondents also had improved identification of correct compression hand position, rate, and depth in performing bystander CPR. This result is meaningful because high-quality CPR is crucial during any resuscitation[20]. Furthermore, there was an increase in the willingness to perform bystander CPR on family members, but a decrease in the willingness to perform CPR on strangers, during the same period.

Rapid access to prehospital emergency medical service, CPR, and rapid facilitation of AED are key factors for improving the survival rate and neurologic outcome of sudden OHCA patients[21–22]. Interval to EMS arrival is between 5–10 minutes and recent Korean reports do not show a significant difference[23–24]. Therefore, performing bystander CPR will be a crucial step in saving OHCA patients. In turn, nationwide and community-wide measures to promote bystander CPR combined with constant monitoring may greatly improve survival and neurological outcomes of OHCA patients.

Studies that are conducted to monitor the current attitude and perception toward CPR in a certain society, such as this one, will help understand the CPR education rate and changes in CPR awareness. Moreover, identifying barriers to performing bystander CPR may contribute to changes in the target society[25–26].

We had already conducted a study regarding CPR willingness[9]. This current study was conducted to not only improve the bystander CPR rate of Daegu citizens, but also to monitor the effects of CPR education in the Daegu area. Previously, we were only able to explain CPR awareness, knowledge, and willingness at a single time point. In this current study, we aimed to compare CPR awareness, willingness, knowledge, and education periodically during a 5-year interval. There are some previous studies based on surveys or interviews[10–13], but it was difficult to find surveys involving trained interviewers conducting face-to-face interviews.

We observed an increase in correct CPR knowledge, which may be influenced by public CPR education efforts. Other studies done by Bull et al. and Nielsen et al. also support this hypothesis, as improved degree of CPR knowledge in a may be associated with public campaigns[7][27]. One of the noticeable finding is that among CPR knowledge, rate of correct answer for chest compression depth and rate was considerably lower than hand position. Other studies comparing CPR skill performance [28–30] reports that rescuers tend to fail more in maintaining chest compression depth or compression rate, compared to keeping correct position of chest compression. This result may be remedied by supplementing CPR education such as using student-directed strategy [31].

Our findings are unique because the marked advancement in CPR knowledge did not seem to contribute to CPR willingness, unfortunately. Willingness to perform bystander CPR on family members increased, but the result was opposite among strangers. This was partially similar to previous studies that report lower willingness to perform CPR on non-family victims compared to family members[9, 32–34]. Many researchers have mentioned that poor CPR knowledge, fear of harming the victim, and possible legal liabilities are key barriers in performing bystander CPR among laypersons[9, 25–26]. We assume that this recent societal trend of not wanting to help others was influenced by the widespread fear of legal consequences. Korean version of Good Samaritan’s Law (Emergency Medical Service Act, Article 5–2)[35] is mainly focused on exemption of any civil or criminal liability when any emergency medical service is provided to patients. Although the law has been under effect since 2008 and more people seem to acknowledge the law based on survey results, this societal trend did not change, but even worsened, after 5 years. In future investigations, this trend should be closely observed and public efforts need to be made in improving this phenomenon.

We can probably assume that OHCAs occurring in individual homes are mainly responded by family members. On the other hand, according to our survey results, people in Daegu area were less willing to perform bystander CPR on strangers, which may lead to a poor outcome for OHCAs occurring in public places. There are some measures proposed recently and require consideration to mediate these obstacles. On CPR education, there are some studies from United Kingdom and Canada [36–37] urging to combine existing CPR education with additional activities to instruct different roles, measure the students’ fear in CPR, and boost confidence. It yielded positive results in overcoming the barriers to bystander CPR.

Aside from CPR education, DA-CPR is one of recently suggested approaches to improving the prehospital CPR rate[38–39]. Considering the barriers in bystander CPR, it is intended for untrained, reluctant, or hesitant laypeople to perform CPR with dispatcher agents encouraging and offering instructions to the caller. The Korean 119 EMS has been implementing DA-CPR since 2012, and the rate of performing CPR through dispatcher assistance has been increasing[4]. Secondly, there is a growing emphasis on first responder programs and rescuer summoning programs in some communities, primarily in the United States and European countries, to promote and enhance bystander CPR[40–41]. In our opinion, those programs should be implemented promptly. A location-based bystander CPR notification system, which was launched in Daegu area only days before the second survey, may play a role implementing those programs. This system can generally alert anyone located near OHCA patients via mobile phone and inform the location of the patient and nearest AED, resulting in faster bystander CPR and defibrillation[42–43]. Thirdly, the current Good Samaritan’s Law in Korea promote bystander CPR by focusing on exemption of any civil or criminal liability, considering that major barriers in performing bystander CPR involves fear of legal consequences. However, this law itself is not well known. Even after 5 years of focusing on improving bystander CPR, only 31.7% of respondents from the second survey recognized this law. The results do not change much even when the entire nation is considered. In a Korean nationwide study conducted in 2012, employees at various workplaces (i.e., those who are usually more interested in news and current issues than students, housewives, or retired people) were surveyed, and only 40.2% ever heard of such legislation[44]. Moreover, the law will not protect laypeople or bystander rescuers from criminal liability if the patient dies. Offering more comprehensive exemption in emergency situations will contribute to better public bystander CPR performance and AED use according to our opinion and that of some other Korean researchers[45–46].

This study has some limitations. Firstly, bias may occur while conducting research using surveys. For example, questions regarding CPR education can lead to more recall bias than using documented records. Respondents may tend to select “morally right” answers, leading to social desirability bias. Selection bias may also be present since we were not able to obtain the exact response rate and the family history of cardiac disease differs significantly between two surveys. Our assumption is that the interviewers were more thoroughly trained in second survey to identify the cardiac diseases and the result may represent more proximate results to actual family history. We also tried to minimize the bias as little as possible by quota sampling and randomization. Secondly, surveying can also lead to disparity in the actual rate of CPR willingness. One may answer willing to perform CPR, but may do nothing in case of actual resuscitation. Although the actual bystander CPR rate in sudden cardiac arrest patients is reported annually by health authorities, it may not properly represent the opinion of entire population. The data also does not report whether the victim was family or stranger to the rescuer, which was important factor in our study. Thirdly, while it is possible to monitor the effect of various interventions in a certain community as a whole, the effect of every particular intervention was not specified, since we could not accurately measure the effect of each intervention. There are studies targeting the effect of individual interventions [7, 27]. Fourthly, we did not include children and adolescents aged <18 years. Globally, in modern CPR education planning, students are considered an important target population because schools are an excellent place to provide early CPR education, which promotes CPR knowledge across generations[47–48]. In fact, our intervention included CPR education in schools and institutions, but the young population had to be excluded from our survey because of restrictions (i.e., requiring parental consent and separate survey questionnaires suitable for children). However, some of adolescents in the time of first survey, may grow up to be adults by the time of second survey. Therefore CPR educations in schools were important aspect in considering as a regional intervention. Lastly, although we segregated respondents based on the target of bystander CPR by relationship, we did not consider special patients (i.e., elderly, young, or pregnant), non-cardiac situations (drowning or traumatic), or CPR method (standard or hands-only), as covered in some studies [49]. We hope to address these issues in future follow-up studies.

In conclusion, we observed improvement in CPR knowledge along with confidence in performing bystander CPR, AED awareness, and frequency, interval, and willingness of CPR education, after the period of national and regional interventions to promote bystander CPR and public CPR education. However, willingness to perform bystander CPR on strangers was decreased. To address this issue, the authors propose promoting noticeable first responder program’s results such as renowned survivors in the community with good neurological outcomes, and cultivating the society with mind that bystander CPR is not futile to the victim but instead helps cure the victim.

## Supporting information

S1 Dataset For answers of two surveys. (SAV)

S1 Text English translation of questionnaire for both surveys. (DOCX)

S1 Table Location of CPR education received by respondents. (DOCX)

S2 Table Reason for not willing to perform bystander CPR and use AED. (DOCX)
