# Supplementary material for: A 5-year change of knowledge and willingness by sampled respondents to perform bystander cardiopulmonary resuscitation in a metropolitan city
Source: PLoS One. 2019 Feb 7;14(2):e0211804. doi: 10.1371/journal.pone.0211804 (PMC6366762; doi:10.1371/journal.pone.0211804)
Supplement: S1 Text — (DOCX) [file pone.0211804.s002.docx]

Supporting Information 2

Questionnaire for first survey, translated in English (Questions only, excluding irrelevant questions for analysis)

1. Select all diseases you have suffered in the past or currently having.

2. Select all diseases your family have suffered in the past or currently having.

4. Did you ever see anyone collapse due to sudden cardiac arrest? If yes, who was it?

5. Have you ever heard of cardiopulmonary resuscitation (CPR)?

6. Do you know how to perform CPR?

7. In your opinion, what is the correct spot to place your hand in chest compression?

8. In your opinion, what is the correct chest compression rate per minute in chest compression?

9. In your opinion, what is the correct chest compression depth in chest compression?

10. Do you feel confident in performing CPR?

11. Will you perform CPR if you come across a cardiac arrest patient who is your family?

12. Will you perform CPR if you come across a cardiac arrest patient who is a stranger to you?

13. If you do not wish to perform CPR, specify the reason.

14. Have you ever heard of an external automated defibrillator (AED)?

15. Did you see any AED installed in public places?

16. Do you know that AED may be used by any layperson?

17. If you come across a cardiac arrest victim and locate an AED nearby, will you use it?

18. If you do not wish to use an AED, specify the reason.

19. What is your occupation?

21. Do you have any CPR education experience? If so, what was the method of education?

21-1. How many times did you receive CPR education?

21-2. When was your latest CPR education?

21-3. Name any institution where you have received CPR education.

1. Hospital ② Korean Association of Cardiopulmonary Resuscitation
2. 1339 Dispatch Center ④ Fire department ⑤ School

⑥ Military ⑦ Workplace ⑧ Reserve Forces Training

⑨ Red Cross ⑩ Public Health Center

⑪ Other ( )

21-4. Did your CPR education include AED training?

22. If you did not receive any CPR training before, what is the reason?

23. Do you wish to receive (further) CPR education in the future?

S4. What is your level of educational degree?

S5. What is your occupation?

Questionnaire for second survey, translated in English (Questions only, excluding irrelevant questions for analysis)

1. Is there any family member suffering from heart diseases? (Angina, myocardial infarction, other cardiac disorders, cardiac surgery/procedure done)

2. Have you ever heard of cardiopulmonary resuscitation (CPR)?

3. Have you ever heard of any heart attack victim revived after CPR via newspaper, television, radio, or other mass media?

4. Do you know how to perform CPR?

6. If you come across this following patient, what is your thought on giving CPR?

- A 50-year old male is unconscious, not responding to your voice. The victim is breathing very shallowly and unusually slow (less than once per 10 seconds).

7. In your opinion, what is the correct spot to place your hand in chest compression?

8. In your opinion, what is the correct chest compression rate per minute in chest compression?

9. In your opinion, what is the correct chest compression depth in chest compression?

10. Do you have confidence in performing CPR?

11. Will you perform CPR if you come across a cardiac arrest patient?

12. If you do not wish to perform CPR, specify the reason.

13. Do you know about the Good Samaritan Law?

14. If there is a service, providing location of cardiac arrest victim near you via text message, will you run to the location and provide bystander CPR?

15. Do you know about the dispatcher assisted CPR?

17. Will you follow the CPR instructions given by dispatcher agents?

18. Have you ever heard of an external automated defibrillator (AED)?

19. Do you know how to use an AED?

20. Did you see any AED installed in public places?

21. If you come across a cardiac arrest victim and locate an AED nearby, will you use it?

22. If you do not wish to use an AED, specify the reason.

23. Do you have any CPR education experience?

23-1. How many times did you receive CPR education?

23-2. When was your latest CPR education?

23-3. Name any institution where you have received CPR education.

1. Hospital ② Korean Association of Cardiopulmonary Resuscitation
2. Fire department ④ School ⑤ Military

⑥ Workplace ⑦ Red Cross ⑧ Public Health Center

⑨ Daegu Metropolitan City Hall ⑩ Other ( )

23-5. Did your CPR education include AED training?

24. Do you wish to receive (further) CPR education in the future?

dq1. What is your level of educational degree?

dq2. What is your occupation?

dq3. How much is your household income in a month?
