# Supplementary material for: A 5-year change of knowledge and willingness by sampled respondents to perform bystander cardiopulmonary resuscitation in a metropolitan city
Source: PLoS One. 2019 Feb 7;14(2):e0211804. doi: 10.1371/journal.pone.0211804 (PMC6366762; doi:10.1371/journal.pone.0211804)
Supplement: S1 Table — (DOCX) [file pone.0211804.s003.docx]

| Place (multiple choices) | 1^st^ survey (%) | 2^nd^ survey  (%) |
| --- | --- | --- |
| Hospital | 4.4 | 5.6 |
| KACPR | 1.2 | 7.3 |
| 1339 Dispatch Center + Fire deparment | 15.9 | 15.7 |
| School | 21.7 | 29.3 |
| Military + Reserve forces training | 17.3 | 16.9 |
| Workplace | 8.5 | 21.6 |
| Red Cross | 3.8 | 7.9 |
| Public Health Center | 0.9 | 3.8 |
| Daegu Metropolitan City | N/A | 8.3 |
| Other | 4.4 | 11.1 |

KACPR: Korean Association of Cardiopulmonary Resuscitation; N/A: not applicable
