# Supplementary material for: A 5-year change of knowledge and willingness by sampled respondents to perform bystander cardiopulmonary resuscitation in a metropolitan city
Source: PLoS One. 2019 Feb 7;14(2):e0211804. doi: 10.1371/journal.pone.0211804 (PMC6366762; doi:10.1371/journal.pone.0211804)
Supplement: S2 Table — (DOCX) [file pone.0211804.s004.docx]

|  | Perform bystander CPR | | Use AED | |
| --- | --- | --- | --- | --- |
| Reason | 1st group^a^ | 2nd group^b^ | 1st group^a^ | 2nd group^b^ |
| Don't know how | 316 (34.2) | 375 (51.5) | 373 (62.1) | 437 (77.5) |
| Fear of harming the victim | 386 (41.8) | 361 (49.6) | 146 (24.4) | 227 (40.2) |
| Not interested in CPR/AED | 10 (1.1) | 9 (1.2) | 47 (7.9) | 12 (2.1) |
| Fear of legal responsibilities | 106 (11.5) | 201 (27.6) | 17 (2.8) | 65 (11.5) |
| Against any CPR/defibrillation by non-medical personnel | 1.8 (2.0) | 9 (1.2) | 13 (2.1) | 12 (2.1) |
| Fear of oral hygiene | 1.0 (1.1) | 4 (0.5) | N/A | N/A |
| Not physically fit to perform CPR | N/A | 11 (1.5) | N/A | N/A |
| Other/no answer | 8 (0.9) | 27 (3.7) | 4 (0.7) | 20 (3.5) |
| Total | 923 | 728 | 600 | 564 |

CPR: cardiopulmonary resuscitation, AED: automated external defibrillator, a: best answer selected, b: multiple answers selected, N/A: not applicable
